# Supplementary material for: The impact of SNP density on quantitative genetic analyses of body size traits in a wild population of Soay sheep
Source: Ecol Evol. 2022 Dec 14;12(12):e9639. doi: 10.1002/ece3.9639 (PMC9750819; doi:10.1002/ece3.9639)
Supplement: Supplementary file 1 — Figure S1. [file ECE3-12-e9639-s001.docx]

Supplementary Figure 1


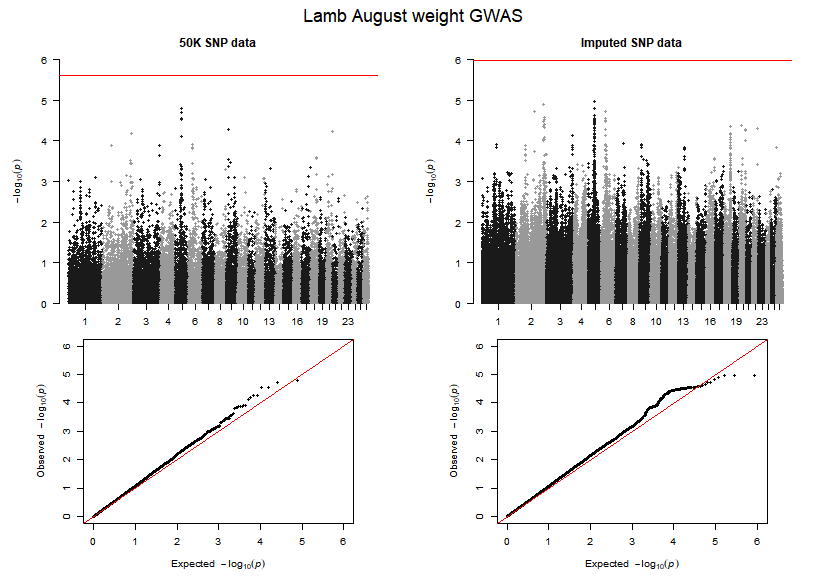

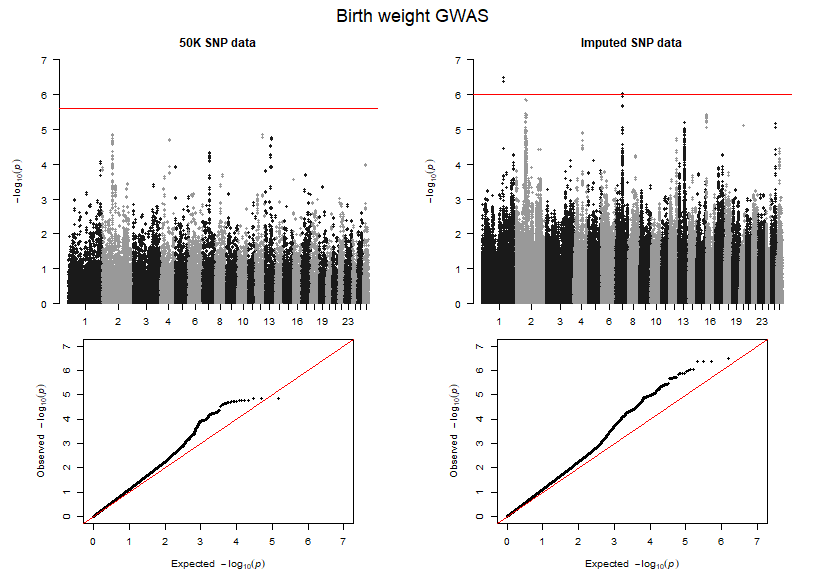


**A**

**B**


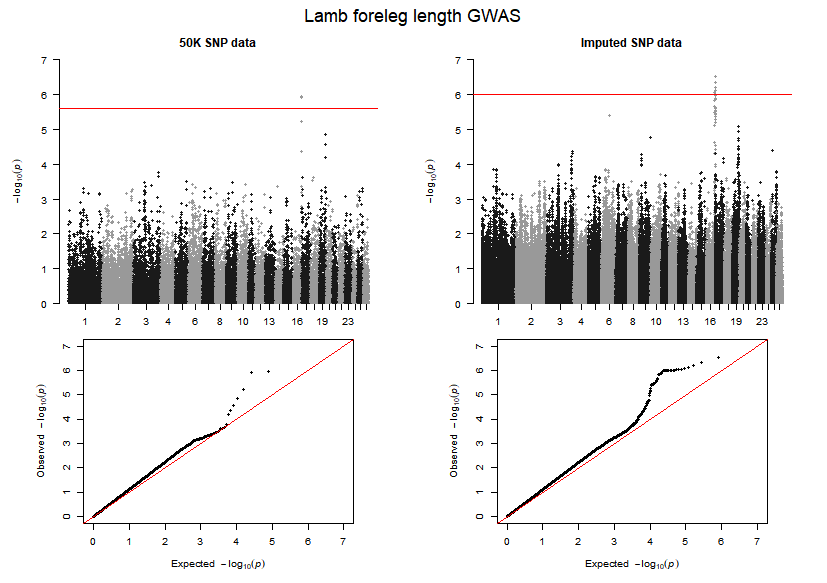

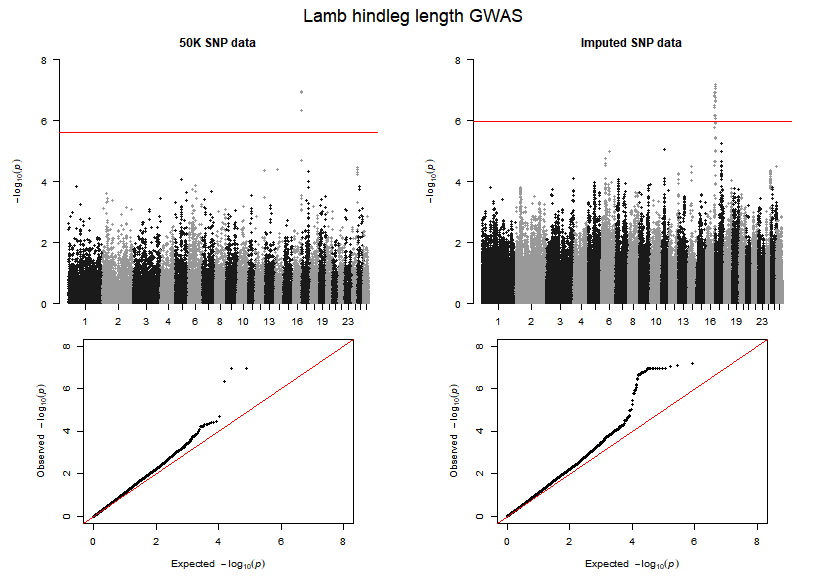


**C**

**D**


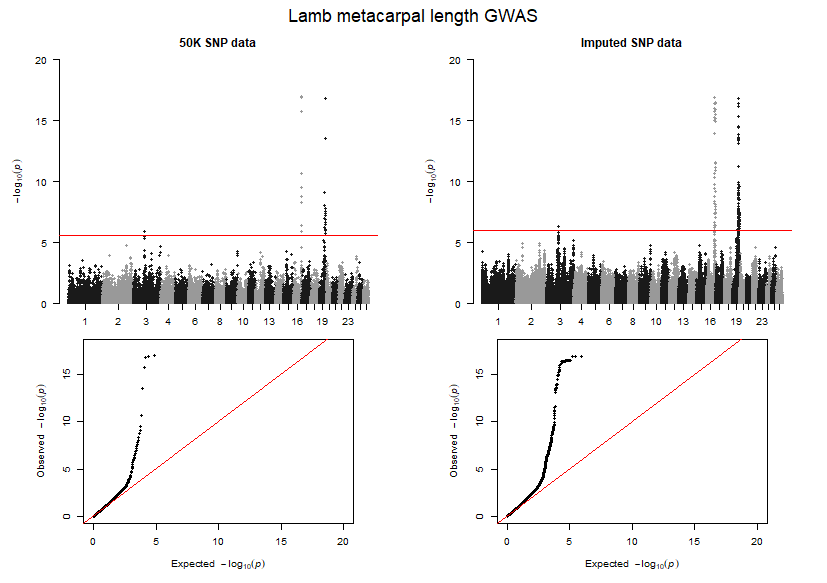

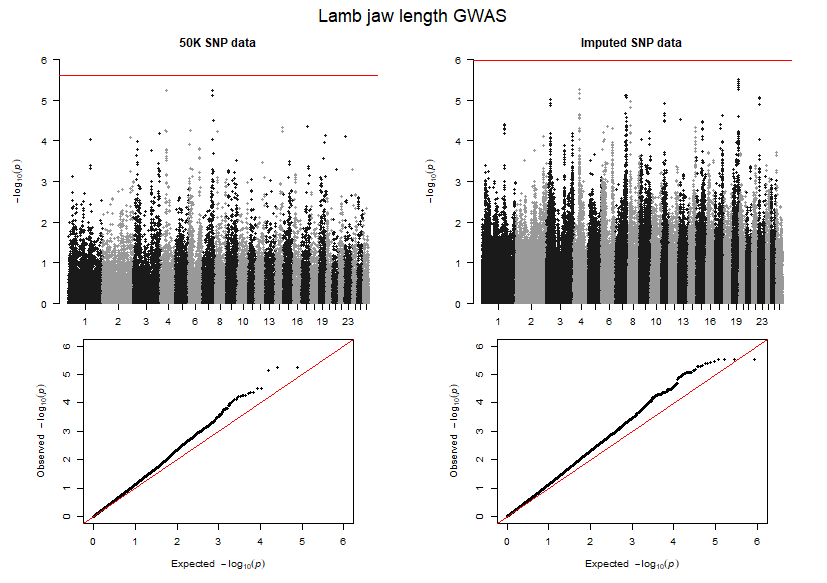


**E**

**F**


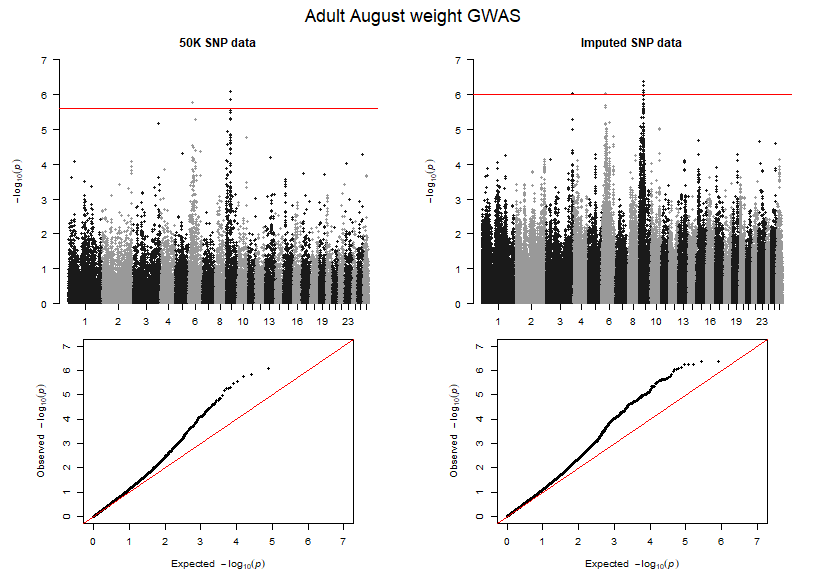

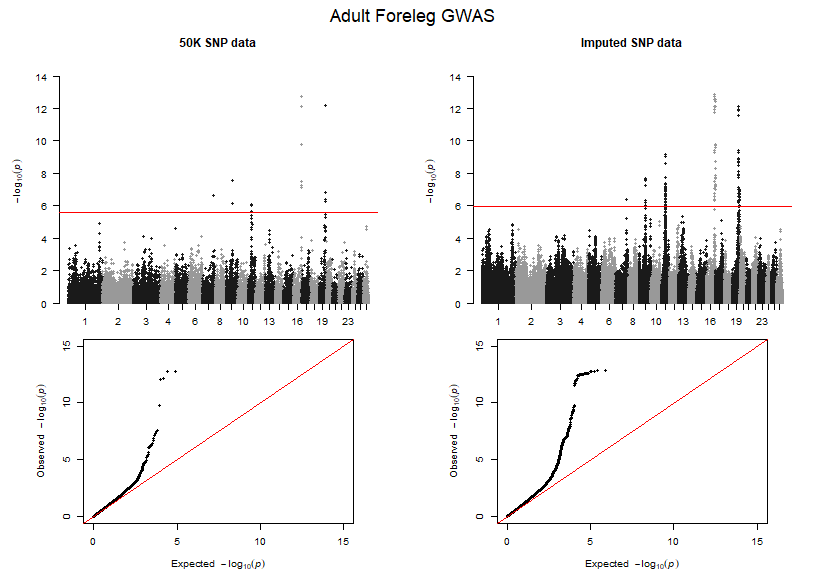


**G**

**H**


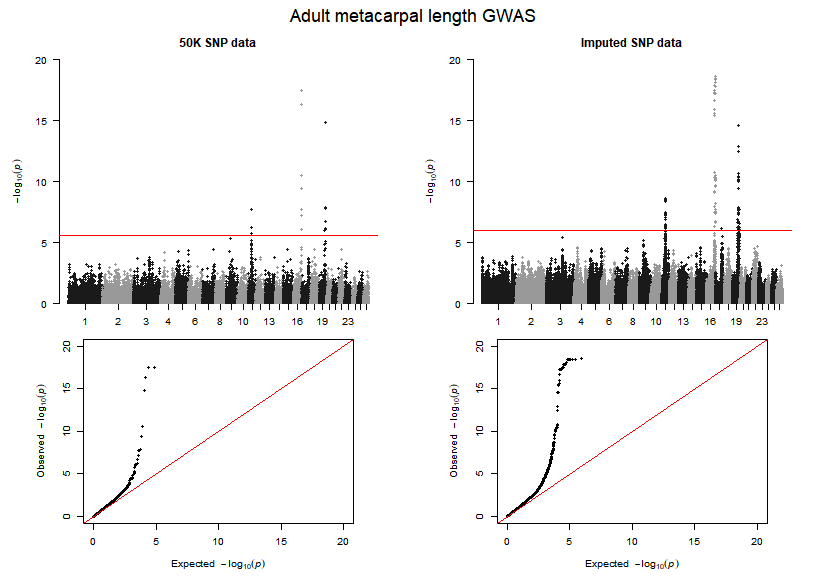

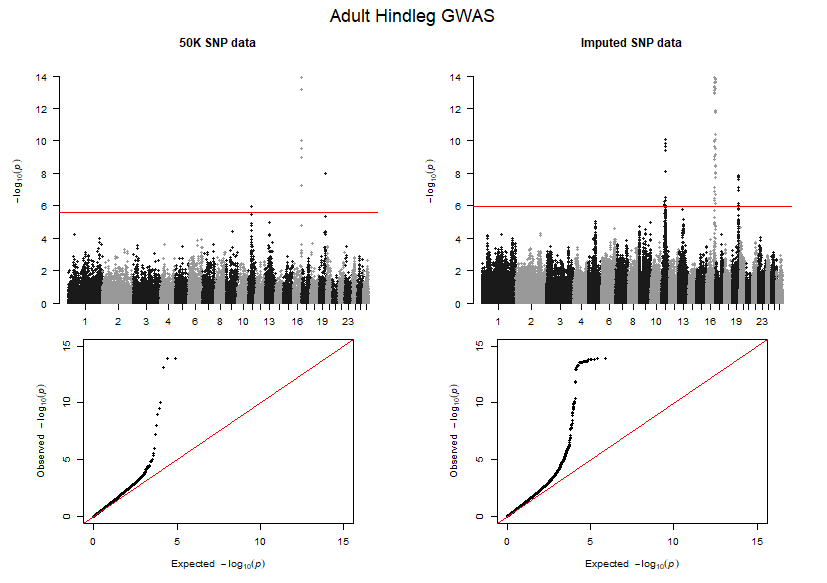


**I**

**J**


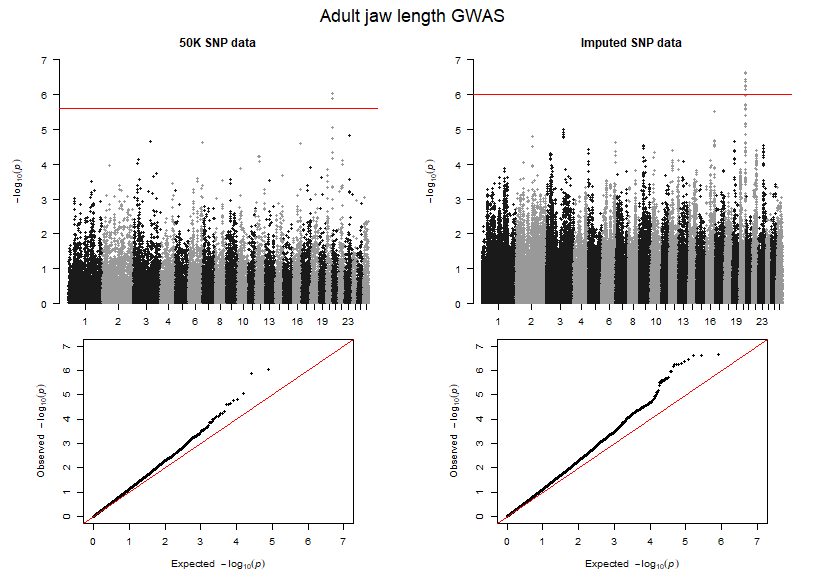


**K**

**Supplementary Figure 1** Manhattan and QQ plots using the 50K SNP data (left) and the imputed SNP data (right) for all traits and age classes. **A)** birth weight, **B)** lamb August weight, **C)** lamb foreleg length, **D)** lamb hindleg length, **E)** lamb metacarpal length, **F)** lamb jaw length, **G)** adult August weight, **H)** adult foreleg length, **I)** adult hindleg length, **J)** adult metacarpal length, and **K)** adult jaw length. In the Manhattan plots, the red line represents the significance threshold (2.49e^-06^ for the 50K SNP data and 1.03e^-06^ for the imputed SNP data) – any SNPs above this threshold are considered to be significantly associated with variation in their respective traits. In the QQ plots, the red line is the X=Y line.
